# Multianalyte liquid biopsy to aid the diagnostic workup of breast cancer

**DOI:** 10.1038/s41523-022-00480-4

**Published:** 2022-09-27

**Authors:** Sonia Maryam Setayesh, Olivia Hart, Amin Naghdloo, Nikki Higa, Jorge Nieva, Janice Lu, Shelley Hwang, Kathy Wilkinson, Michael Kidd, Amanda Anderson, Carmen Ruiz Velasco, Anand Kolatkar, Nicholas Matsumoto, Rafael Nevarez, James B. Hicks, Jeremy Mason, Stephanie N. Shishido, Peter Kuhn

**Affiliations:** 1 Convergent Science Institute in Cancer, Michelson Center for Convergent Bioscience, Dornsife College of Letters, Arts and Sciences, University of Southern California, Los Angeles, CA 90089 USA; 2 Norris Cancer Center, Keck School of Medicine, University of Southern California, Los Angeles, CA 90033 USA; 3 Department of Surgery, Duke University School of Medicine, Durham, NC 27710 USA; 4 Billings Clinic, Billings, MT 59107 USA; 5 Epic Sciences, San Diego, CA 92121 USA

**Keywords:** Breast cancer, Cancer screening, Cancer

## Abstract

Breast cancer (BC) affects 1 in every 8 women in the United States and is currently the most prevalent cancer worldwide. Precise staging at diagnosis and prognosis are essential components for the clinical management of BC patients. In this study, we set out to evaluate the feasibility of the high-definition single cell (HDSCA) liquid biopsy (LBx) platform to stratify late-stage BC, early-stage BC, and normal donors using peripheral blood samples. Utilizing 5 biomarkers, we identified rare circulating events with epithelial, mesenchymal, endothelial and hematological origin. We detected a higher level of CTCs in late-stage patients, compared to the early-stage and normal donors. Additionally, we observed more tumor-associated large extracellular vesicles (LEVs) in the early-stage, compared to late-stage and the normal donor groups. Overall, we were able to detect reproducible patterns in the enumeration of rare cells and LEVs of cancer vs. normal donors and early-stage vs. late-stage BC with high accuracy, allowing for robust stratification. Our findings illustrate the feasibility of the LBx assay to provide robust detection of rare circulating events in peripheral blood draws and to stratify late-stage BC, early-stage BC, and normal donor samples.

## Introduction

Accurate prognosis at the time of a diagnosis with early-stage breast cancer is a critical aspect of the diagnostic workup. Analytes in the blood-based liquid biopsy carry the opportunity for better characterization of the systemic burden of the disease during this clinical process. Breast cancer (BC) is the most common cancer in women globally and with 7.8 million cases diagnosed in the past 5 years, it is the world’s most prevalent cancer overall^1–3^. Approximately 94% of patients are initially diagnosed with early-stage BC, without evidence of macroscopic metastasis, however, despite the initial lack of detectable metastases and administration of subsequent treatments, 40% of the early-stage BC patients will go on to develop recurrence over their lifetime^4–9^. Relapse, progression, and onset of distant metastasis (late-stage BC) have a significant negative impact on clinical outcomes, dropping the 5-year survival rate from 91% to less than 30%^1,3^. Considering the impact on survival rates, it is vital that robust stratification of early-stage BC be made possible at the time of the initial diagnostic workup and throughout the course of the disease.

Currently, the standard screening method for BC is mammography, with a tissue biopsy to confirm diagnosis^3,4^. In patients with biopsy confirmed cases of BC, tumor burden and treatment response are typically assessed by clinical evaluation of symptoms alongside imaging^4^. While cross sectional advanced imaging is sometimes used to identify disease spread, it is expensive, often inconclusive, and fails to provide insight into the status and changes of the molecular profile of the tumor. Solid tissue biopsies have great utility in clinical care and can provide information on tumor biomarker and histological subtyping, molecular profiles, and advise treatment planning. Nevertheless, they have several caveats. First, primary tumors or metastatic lesions are not always easily accessible. Second, although solid biopsies provide valuable insights into the molecular signatures of the tumor, they are limited to the precise sampling area and could fail to capture the tumor heterogeneity^10–14^. However, since CTCs have been shown to be shed from both primary and metastatic tumor sites, they have demonstrated the potential to resolve spatial heterogeneity of the tumor^15–21^. Third, and most crucial, solid biopsies are inherently incompatible with characterization of the subclinical systemic spread of the disease in addition to being challenging for longitudinal monitoring since they are painful, invasive, and always carry a potential risk to the patient^22–26^.

Liquid biopsy (LBx), with a focus on peripheral blood, is a minimally-invasive method that can provide key information about the tumor and the systemic burden of the disease in the circulatory system^27,28^. The utility of LBx for BC detection in the metastatic setting has been well-established with numerous clinical trials focusing on their utility to inform clinical decision-making and improve patient outcomes^29–35^. Most of the LBx studies on BC focus on the presence of circulating tumor cells (CTCs), however, in the case of early-stage BC where CTC positive patients are scarce^36–40^, more comprehensive analysis of tumor-related analytes in the LBx could be beneficial to assess the disease status. Currently, the CellSearch (Menarini Silicon Biosystems, Bologna, Italy) system has 510k device clearance by the FDA for BC and is limited only to late-stage metastatic disease^41^. CellSearch enriches for circulating tumor cells (CTCs) using the cell surface marker Epithelial Cell Adhesion Molecule (EpCAM), which makes it unable to detect cells with downregulated EpCAM undergoing epithelial-to-mesenchymal transition (EMT) and mesenchymal CTCs. With the growing focus on mesenchymal CTCs and their more aggressive role as metastatic precursors compared to epithelial CTCs^42,43^, there is a need for next generation LBx systems that can detect the more complete set of epithelial, mesenchymal, endothelial and transitional cell types.

The third generation high-definition single cell assay (HDSCA3.0) workflow provides the opportunity to identify and characterize epithelial, mesenchymal, endothelial, and hematopoietic cells, as well as large extracellular vesicles (LEVs), building a platform capable of providing a more comprehensive overview of the circulating rare events and capturing the heterogeneity of the LBx^44^. The non-enrichment method of HDSCA provides a single cell profile of all circulating events, with a sensitivity of 1 in 6 million cells, compared to clinical flow cytometry, which has a reported sensitivity of 10^−3^ to 10^−5 ^^45^. Furthermore, the HDSCA workflow samples do not require immediate analysis after processing and can remain in cryopreservation for prolonged periods prior to analysis, as opposed to other methodologies which typically requires immediate analysis. Last, by combining high resolution imaging and immunofluorescence, we can capture a higher resolution of cellular morphology and biomarker localization.

In this study, we demonstrate the feasibility of using the HDSCA3.0 to stratify late-stage BC, early-stage BC, and normal blood donor status, using peripheral blood samples. We observe a distinctly higher presence of CTCs in the late-stage BC, compared to the early-stage and normal groups. Additionally, we determine that tumor-associated LEVs are found more frequently and in greater abundance in the early-stage BC group compared to late-stage and normal blood donor groups. In combination, this allows for both the stratification of cancer vs. normal and early- vs. late-stage BC with statistical confidence. Our results open the opportunity for a complementary LBx at the time of diagnostic workup for cancer detection, stage stratification, and disease monitoring.

## Results

### Patient demographics and clinical baseline

A total of 155 blood draws from 130 participants, with 74 (56.9%) treatment-naive, nonmetastatic early-stage patients, 26 (20%) metastatic late-stage, and 30 (23.1%) normal donors, were included in this study. All participants were female. Patients’ demographics are provided in Supplementary Table 1. The total sample set included 310 slides each containing approximately 3 million nucleated cells that were processed and analyzed for rare event detection (Methods).

### Identification, enumeration, and morphometric analysis of rare cells

**Fig. 1:**
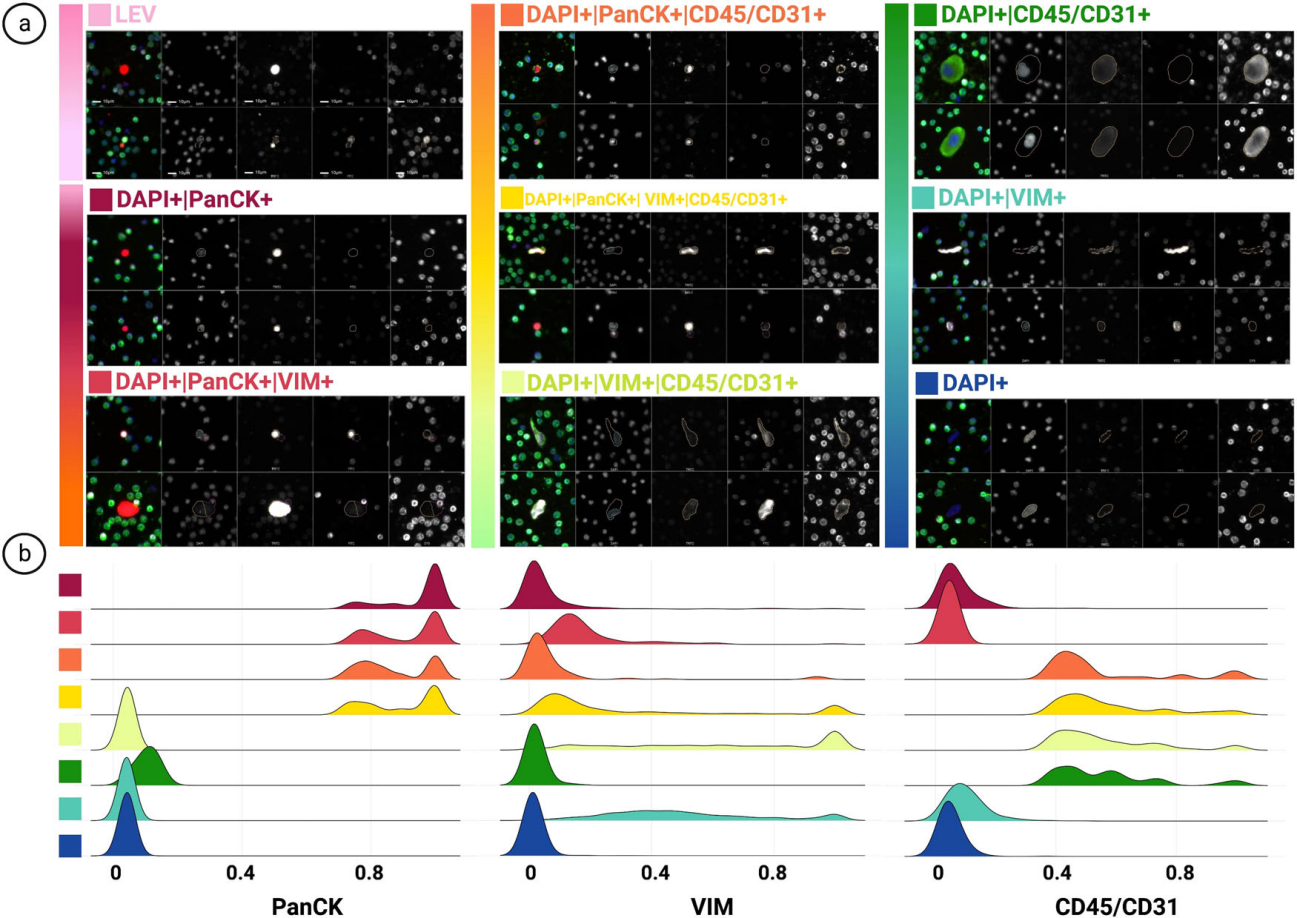
HDSCA3.0 Rare Event Gallery. **a** Images represent two candidate rare events, categorized by marker expression. **b** Signal distribution of immunofluorescent markers for channel-classified cells. Designated colors represent each channel-classified group, assigned in **a**. (LEVs not included due to variation in segmentation). Scale bar represent 10 μm.

**Fig. 2:**
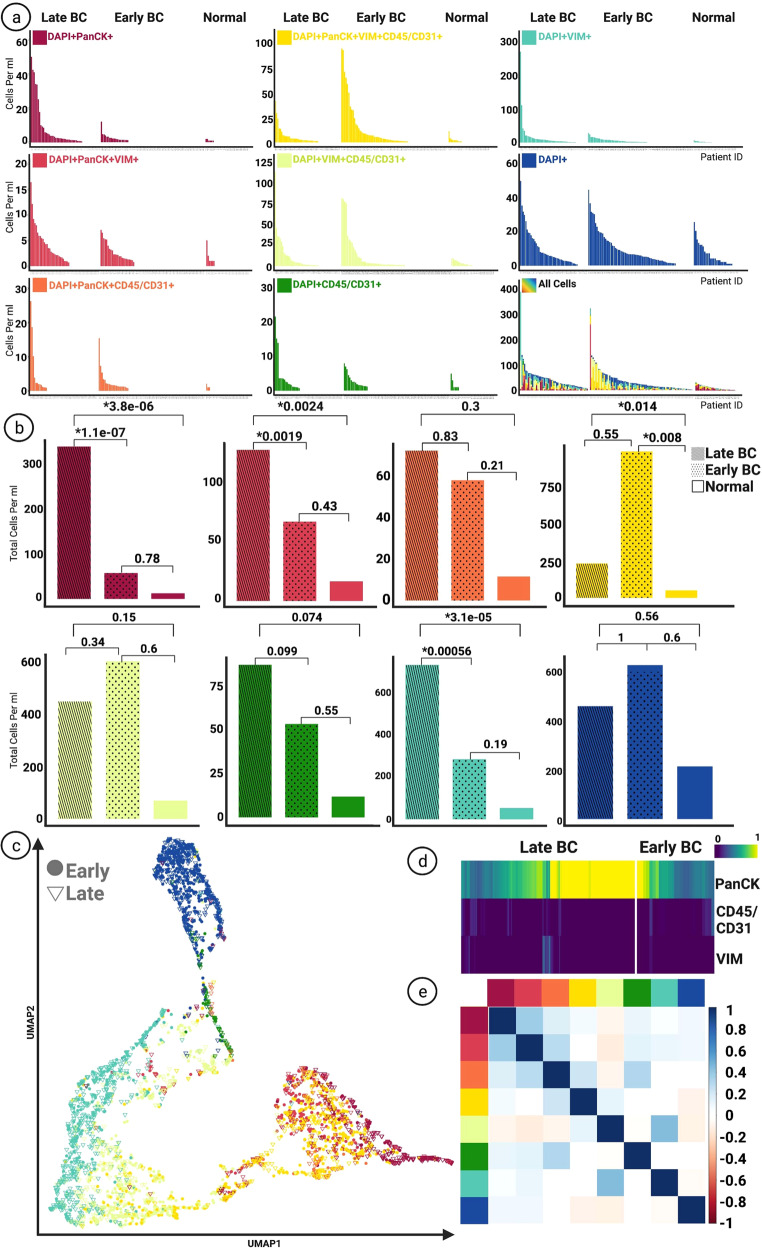
Enumeration of Circulating Rare Cells. **a** Frequency of enumerated rare cells between late-stage, early-stage, and the normal group based on channel classification. **b** Comparison of the distribution of rare cells between groups, Kruskal-Wallis H test (one-way ANOVA) performed on all samples. Graphs display total cells per ml. All *p* values below *0.05 considered statistically significant. **c** UMAP rendering of rare cells based on morphometric features. Each designated color represents a classification group marked in **a**. **d** Heatmap illustrating signal intensity of biomarkers on DAPI + | PanCK+ cells detected in late-stage and early-stage BC groups. **e** Correlation plot (Pearson correlation) between rare cell categories and LEVs for all samples. Each designated color represents a classification group marked in the figure on panel **a**.

We identified and categorized candidate rare cells using an automated rare cell detection workflow followed by manual enumeration based on the four-channel immunofluorescence staining corresponding to DAPI, PanCK, VIM, CD45/CD31, and cellular morphology (Fig. 1). Kruskal-Wallis H test (one-way ANOVA) was performed for all comparisons and the *p* values below *0.05 were considered statistically significant. Enumeration of total rare cells revealed a significantly higher overall count in late-stage BC patients (mean = 48.67, median = 36.36, range = 8.01–383.32 cells/ml) compared to early-stage BC (mean = 36.19, median = 23.06, range = 1.58–284.54 cells/ml; *p* = 0.01), and late-stage BC compared to normal donors (mean = 14.27, median = 12.89, range = 0-37.43 cells/ml; *p* = 0.0015×10^04^). A significant difference was also observed between the early-stage BC patients and normal donors (*p* = 0.0012) (Fig. 2a, b).

CTCs that were identified as DAPI + | PanCK+ were defined as epi.CTCs and enumerated for normal donor, early-stage BC, and late-stage BC samples. The epi.CTC enumeration of all samples revealed a median of 0 cells/ml (mean = 2.66, range = 0–50.10 cells/ml). For the late-stage group, 75% of patients had at least one epi.CTC (mean = 6.75, median = 2.02, range = 0–50.10 cells/ml), compared to only 27% of early-stage patients (mean = 0.77, median = 0, range = 0–12.13 cells/ml; *p* = 0.0011×10^−04^). Late-stage patients had a significantly higher level of epi.CTCs than the normal donor group (mean = 0.39, median = 0, range = 0–2 cells/ml; *p* = 0.0038×10^−03^). No significant difference in the epi.CTCs was observed between the early-stage BC and the normal donor groups. (Fig. 2a, b).

VIM + CTCs (mes.CTCs) were identified as DAPI + | PanCK + |VIM + . For all samples we observed a median of 0 cells/ml (mean = 1.27, range =0–16.42). The late-stage BC group revealed a significantly higher overall count of mes.CTCs (mean = 2.52, median = 1.02, range = 0–16.42 cells/ml), in comparison with the early-stage BC (mean = 0.91, median = 0, range = 0–7.06 cells/ml; *p* = 0.0019) and the normal donor (mean = 0.55, median = 0, range = 0-5 cells/ml; *p* = 0.0024) groups. No significant difference was observed between the normal donor and early-stage BC groups) (Fig. 2a, b).

Additional candidate CTCs include PanCK + |CD45/CD31 + (double positive CTC) and PanCK + |VIM + | CD45/CD31 + (triple positive CTC) cells. No significant difference was observed between the levels of double positive CTCs between the groups. The triple positive CTCs were found at significantly higher frequencies in both the early-stage BC (mean = 12.80, median = 1.80, range = 0–240.04 cells/ml; *p* = 0.008) and the late-stage BC (mean = 4.34, median = 2.07, range = 0–40.56 cells/ml; *p* = 0.014) compared to the normal donor (mean = 1.56, median = 0, range = 0–17.062 cells/ml) group. No significant difference was observed in the comparison between the early- and late-stage groups (Fig. 2a, b).

Other detectable rare cells include morphologically distinct VIM + | CD45/CD31 + | DAPI + , CD45/CD31 + | DAPI + , DAPI + , and VIM + | DAPI + cells. The VIM + | DAPI + only cells showed a significant increase in the late-stage group (mean = 14.43, median = 4.74, range = 0–266.82 cells/ml), compared to the early-stage (mean = 3.84, median = 1.44, range = 0–27.81 cells/ml; *p* = 0.00056) and the normal donor (mean = 1.72, median = 0.93, range = 0–12.10 cells/ml; *p* = 0.0031×10^−02^) groups (Fig. 2a, b).

Morphological analysis was conducted on the identified rare cells based on extracted image features from EBImage. A visual representation of the identified rare cells based on their morphometric features has been provided as a uniform manifold approximation and projection (UMAP) figure (Fig. 2c), as well as a low-dimensional TSNE plot (Supplementary Figure 1). In the UMAP projection, the majority of manually classified cells cluster together by channel type classification, indicating robust manual classification across the cohort. The CTCs detected in late-stage BC samples demonstrated higher PanCK expression, measured by normalized signal intensity, (mean = 0.80, median = 0.87, range = 0–0.60) than their early-stage BC counterparts (mean = 0.61, median = 0.56, range = 0.44–0.74, *p* = 0.00015) (Fig. 2d).

A correlation analysis between the frequency of classified rare cell categories was conducted for all samples and no strong correlation was found (Fig. 2e).

### Identification and enumeration of tumor-associated LEVs

**Fig. 3:**
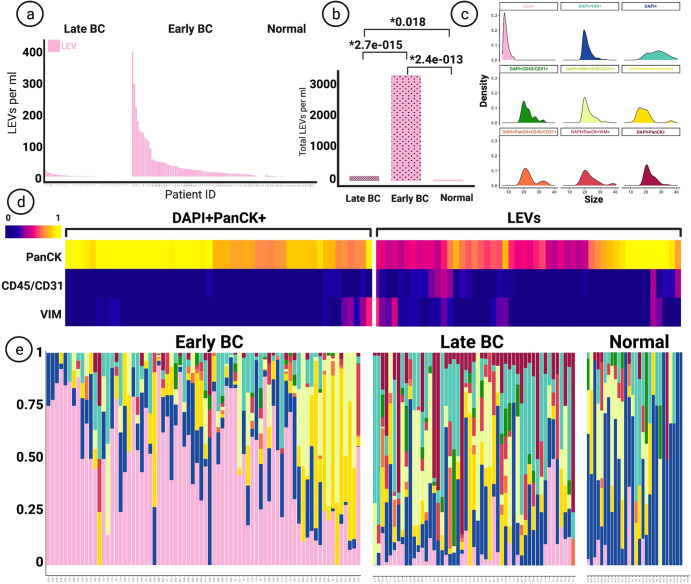
Comparison of Tumor-Associated LEVs. **a** Frequency of enumerated LEVs between late-stage, early-stage, and the normal group based on channel classification. **b** Comparison of the distribution of LEVs between groups, Kruskal-Wallis H test (one-way ANOVA) performed. All *p* values below *0.05 considered statistically significant. **c** Size comparison of LEVs and rare cell events. All sizes represent diameters in micron. Sizes calculated by feature conversion from 100x images. **d** Heatmap displaying signal intensity of biomarkers on LEVs and DAPI + PanCK+ cells. **e** Scaled frequency plots of rare cells and LEVs in patients, designated colors represent classification groups marked in the figure on panel **c**.

LEVs, classified as DAPI- | PanCK+ events were most prevalent in the early-stage BC group, with 94% of patients having at least one LEV per ml, compared to 60% in the late-stage group (Fig. 3a). Kruskal-Wallis H test (one-way ANOVA) was performed and all *p* values below *0.05 were considered statistically significant. The frequency of LEVs was overall elevated in the early-stage BC group (mean = 43.78, median = 20.31, range = 0–400.52), compared to the late-stage BC (mean = 2.92, median = 1.37, range = 0–21.91, *p* = 0.0027×10^−012^) and the normal donor (mean = 0.99, median = 0, range = 0 to 6.73, *p* = 0.0024×10^−10^) groups. A significant difference was also observed between the late-stage BC and the normal donor groups (*p* = 0.018) (Fig. 3b). Identified LEVs fell into the size range (5.89–14.02 micrometer in diameter), representing the smallest rare event category (Fig. 3c). The marker expression profile of classified LEVs were similar to that of epi.CTCs, with some expression of VIM and CD45/CD31 detected, as shown in Fig. 3d. Scaled plots depicted in Fig. 3e indicate a higher overall presence of LEVs in the early-stage group, compared to the late-stage and normal donor. A correlation analysis between the frequency of classified rare cell categories and LEVs was conducted for all samples and no strong correlation was found.

### Correlations with clinical outcome

In the patient population with identified hormone receptor (HR) and end-of-therapy status (44 early-stage/57% and 12 late-stage/46%) (Supplementary Table 1), we evaluated whether the identified rare events are associated with clinical markers and patient outcomes. In the early-stage BC group, the overall median time from diagnosis to follow-up was 27 months (range = 8 to 99, n = 44), with no reported mortalities. We performed Kruskal-Wallis H test (one-way ANOVA) and all p values below *0.05 were considered statistically significant.

**Fig. 4:**
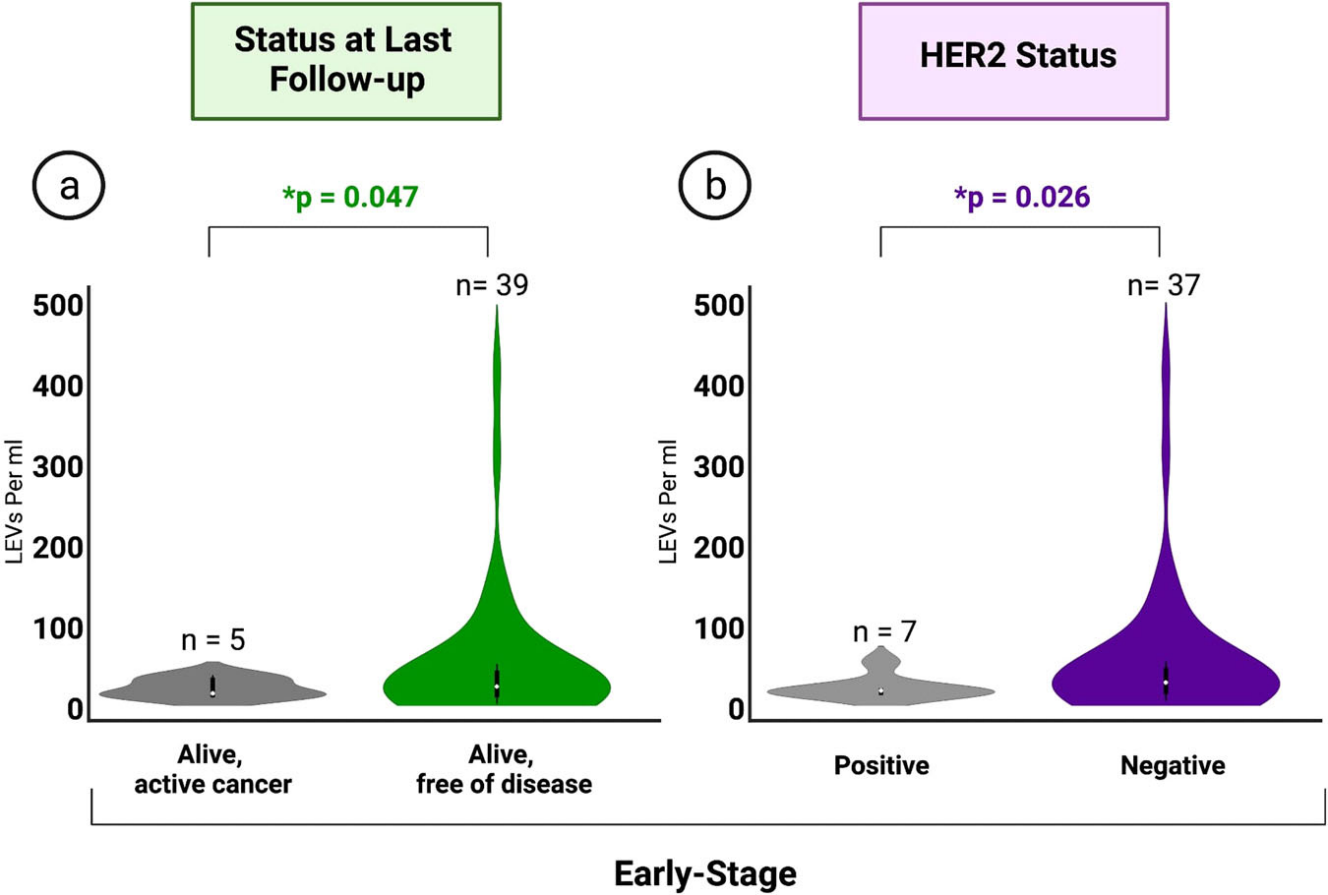
Clinical Data. **a** Comparison of summed LEV levels between differing statuses at last follow-up in early-stage BC. Kruskal-Wallis H test (one-way ANOVA) performed. All *p* values below *0.05 considered statistically significant. **b** Comparison of summed LEV levels between early-stage patients with clinically identified HER2 + and HER2- tumors. Kruskal-Wallis H test (one-way ANOVA) performed. All *p* values below *0.05 considered statistically significant. Data illustrated in truncated violin plots.

Our results indicate a significantly higher frequency of LEVs in the early-stage BC group with the last follow-up status of “alive, free of disease” (mean = 46.10, median = 20.25, range = 0–400.52 LEVs/ml, *n* = 39) in comparison to those with “alive, active cancer” (mean = 18.03, median = 11.89, range = 7.41–32.88 LEVs/ml; *p* = 0.047, *n* = 5) (Fig. 4a). Levels were also found to be elevated in patients with human epidermal growth factor receptor 2 (HER2) negative (mean = 48.22, median = 21.46, range = 0–400.52 LEVs/ml, *n* = 37) compared to HER2 positive (mean = 15.13, median = 11.34, range = 7.41–46.41 LEVs/ml; *p* = 0.026, *n* = 7) tumor status (Fig. 4b). No significant correlation was observed between HER2 tumor status and follow-up patient status in the early-stage BC patients.

In the late-stage BC group, the overall median time from diagnosis to follow-up was 19.5 months (range = 1 to 41, *n* = 14), with no cases reported to be cancer-free. We found significantly higher epi.CTC levels in group with the follow-up status of “deceased, active cancer on day of death” (mean = 21.96, median = 17.68, range = 0 to 50.10 cell/ml, *n* = 6), compared with “alive, active cancer” (mean = 1.37, median = 1.48, range = 0–3.40 cell/ml; *p* = 0.045, *n* = 8).

Epi.CTC counts were also found to be elevated in BC patients with estrogen receptor (ER) positive (mean = 14.78, median = 2.18, range = 0–50.10, *n* = 9) compared to ER negative (mean = 1.93, median = 2.44, range = 0–3.83, *p* = 0.072, *n* = 5) tumor status. The same relationship was also detected between the progesterone receptor (PR) positive (mean = 20.33, median = 13.70, range = 0–50.10, *n* = 6) and PR negative (mean = 2.59, median = 1.69, range = 0–10.15, *p* = 0.086, *n* = 8) patients, although both levels did not reach statistical significance. No significant relationship was observed between ER/PR tumor status and follow-up patient status in the late-stage BC patients. No significant difference was observed between HER2 tumor status and epi.CTC levels.

### Patient level classification model

**Fig. 5:**
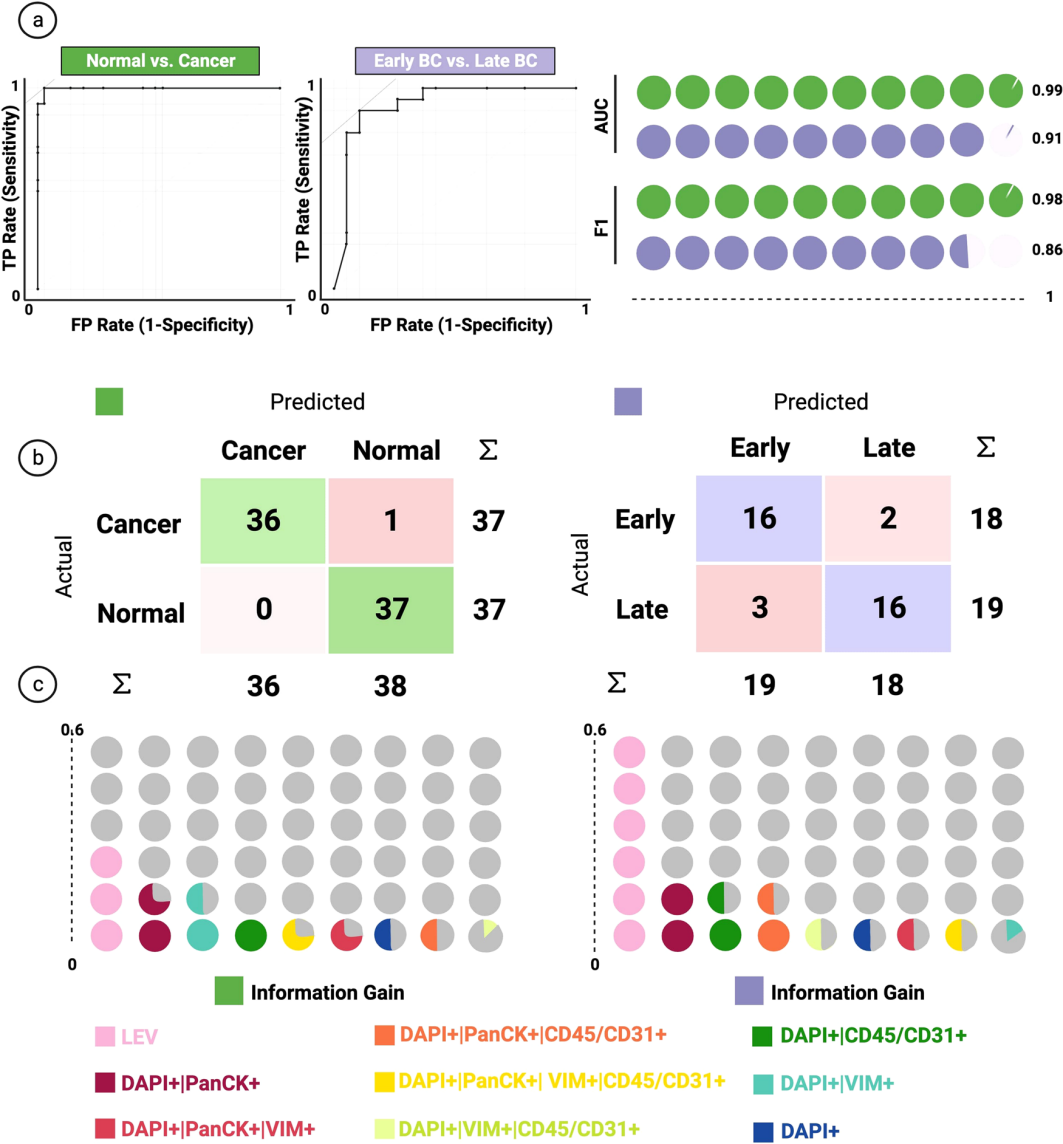
Classification Model. **a** On the left, ROC analysis of the random forest model for each target variable class. Curves represent merged prediction from folds. On the right, AUC and F1 score of the corresponding models. **b** Confusion matrix of the random forest model on the test set. **c** Ranking of the features for classification based on information gain. Each color represents a channel-classified event group detailed marked in the figure.

The random forest model exhibited acceptable performance, as measured by the ROC/confusion matrix, between normal vs. cancer and early-stage vs. late-stage comparisons (Fig. 5a, b). LEV enumeration was the strongest predictor for correctly classifying into late, early, and normal, followed by epi.CTC enumeration. (Fig. 5c). Our normal vs. cancer model reached 0.99 AUC in classification and an F1 score (0.98%), exhibiting robust performance. Additionally, our early-stage vs. late-stage model reached 0.91 AUC, with similar performance for F1 score (0.86%) (Fig. 5d).

## Discussion

In this study, we set out to stratify late-stage BC, early-stage BC, and normal donor peripheral blood samples based on rare circulating events identified using the HDSCA3.0 LBx platform. We utilized 5 biomarkers to identify and distinguish rare circulating events as epithelial, mesenchymal, endothelial, or hematological origin. Using this comprehensive profiling without prior enrichment, we were able to observe events in all samples, allowing for robust stratification with both manual classification and mathematical model-building approaches. We were able to detect reproducible patterns in the enumeration of rare cells and LEVs. These reproducible patterns separate the relevant groups of cancer vs. normal control and early-stage cancer vs. late-stage cancer with high accuracy. Our findings demonstrate the feasibility to provide robust and reproducible detection of rare circulating events in peripheral blood draws and to stratify late-stage BC, early-stage BC, and normal donor samples.

Since metastasis is the most common cause of cancer mortality^1^, earlier detection and precise diagnosis of existent and early tumor dissemination is imperative to improving patient outcomes. In our study, we found a statistically significant increase of CTCs in patients of the late-stage compared to early-stage BC groups. Previous studies have attributed the higher frequency of CTCs in late-stage BC patients to the dissemination of tumor^46^, therefore the lower incidence rate observed in the early-stage cancer setting could be explained by the organ-confined nature of the disease and lack of widespread metastasis. Previous work has demonstrated a link between CTC burden in late-stage BC and progression-free survival^47^, however, administration of treatment has been shown to affect the abundance of CTCs^48^. In this study of late-stage BC patients, with draws taken either on and off therapy, we were able to detect epi.CTCs in 75% of the samples and observe negative association of epi.CTC count with overall survival. Therefore, our results using a high-sensitivity nonenrichment technology demonstrate that epi.CTCs may still be detected, and provide prognostic value prior to the initiation of therapy, as well as during treatment. Furthermore, we have observed heterogeneity in the channel-type classification of rare cells, and each is likely a mixture of multiple functional cell types. For the PanCK + |CD45/CD31 + population, we have previously identified this group as platelet-coated CTCs^44^. The group of PanCK + |VIM + | CD45/CD31 + cells that display platelet coating around the cell can be mesenchymal platelet-coated CTCs, as previously described^44^. Additionally, bona-fide PanCK + |VIM + | CD31 + cells can show up triple positive with a distinctive morphology that is characteristic of the endothelial-phenotype^49^.

Despite advances in the LBx field, the low abundance of CTCs, especially in early-stage cancer, remains a challenge for establishing precise diagnosis and prognosis in this setting. Furthermore, tumors are complex and are comprised of heterogeneous cell types, with CTCs that are defined by dual positivity for EpCAM and Cytokeratin only representing a fraction of the total tumor cells responsible for dissemination and relapse^50–52^. Motivated by these prior observations, this next-generation LBx was designed to identify and characterize the tumor heterogeneity in the circulatory system. By including eight rare cell categories, we were able to observe the heterogeneous phenotypes in circulation and to use these multiple LBx analytes to stratify the samples according to disease status with high statistical significance.

Detection of LEVs represent a promising new LBx analyte^53^. Our results demonstrate a statistically higher overall presence of tumor-associated LEVs in the early-stage BC group, compared to the late-stage BC group and the normal donors. The high level of LEVs in the early-stage BC patients could be explained by the presence of the primary tumor, since these early-stage BC patient samples were collected prior to any treatment, at which time the patient still had their primary tumor intact. This contrasts with the late-stage patients, who are more likely to have had their primary tumor removed prior to the time of blood draw. Tumor-associated LEVs have been described as a component of the tumor microenvironment^54^, and primary tumors have been shown to harbor more cellular heterogeneity in comparison to metastatic lesions which are mostly composed of tumor cells^55^. Additionally, previous findings have implicated extracellular vesicles for their role in facilitating premetastatic niche preparation^56,57^. Tumor progression and metastasis requires the acquisition of invasive traits within the primary tumor alongside the generation of a permissive microenvironment at distant metastatic sites. Previous studies have found that in the case of BC, extracellular vesicles can initiate organ-specific premetastatic niche preparation^58^. These results suggest that there is an additional possibility that LEVs are secreted into circulation in pre-metastatic early-stage disease from the primary tumor to facilitate the preparation of metastatic niches and are less inclined to be present in late-stage disease where the metastatic sites are well-established. Furthermore, a large number of tumor-derived vesicles have been shown to induce immune cell dysfunction and increase immunosuppression to promote tumor progression and immune escape^59–61^. Tumor-derived extracellular vesicles can impact tumor immunity by impairing antigen presentation^62^, inhibiting cytotoxic immune cell activity^63–65^, or increasing immunoregulatory activity^66,67^. In the present study, the finding of the high prevalence of large EVs in the treatment-naive breast cancers may be indicative of biology distinct from the late-stage observations and model findings. Our study demonstrates that detection of LEVs, when applied alongside rare cell enumeration, provides a more sensitive and specific LBx analysis. Future experiments in both clinical samples and model systems will be needed to further delineate the exact mechanism of LEV interactions with the immune system as well as their role in metastatic niche preparation.

The OPTICOLL study was originally designed to provide a comprehensive analysis of pre-analytical variables of LBx^68,69^ and is providing a platform for discovery using sample preparation methods that have been previously validated. A limitation of this study is the number of patients with the sufficient follow-up that we were able to include. The results of this study should however provide sufficient feasibility to conduct larger trials and higher patient recruitment as the next step towards clinical utility. Both the use of additional lineage markers and the inclusion of LEVs in addition to CTCs has significantly advanced our ability to separate the patient groups. The patients with sufficient follow-up did not yet include plasma preparation for cell-free analysis, which one would expect to also add value. However, previous studies in breast cancer have shown that cfDNA is not able to stratify breast cancer stages in suspected patient populations, in treatment-naive samples^70^. Additionally, for early-stage BC, there is a challenge due to the lower concentrations of ctDNA, compared to cfDNA^71^. In future studies, we plan to further investigate the merits of a combined approach for analysis as the sample preparation has now been optimized to enable both, cell-free and cell-based analysis from the same blood draw.

However, despite the current limitations, we were able to observe a highly significant difference in the LBx analytes between breast cancer patients and normal controls, and between the late-stage and early-stage BC samples collected. While the current observations are consistent with prior hypotheses of various liquid biopsy analytes, we expect these results will trigger further model system experiments to continue the exploration of the early and late-stage implications of LEVs in particular as well as the design of additional trials to define the clinical utility as a potential adjunct to the diagnostic workup.

A more comprehensive profiling of the LBx as demonstrated here has the potential to complement the current diagnostic workup following a positive screening test. The current NCCN guidelines do not recommend systemic imaging such as FDG-PET scanning for the majority of early-stage patients as most patients will receive some form of adjuvant treatment^72^. However, LBx findings, such as the frequencies of LEVs and CTCs, may provide diagnostic and prognostic information that would impact the utility of adjuvant systemic therapy in subsets of patients. Emerging data has also shown the importance of tumor profiling in the recurrence setting for optimized intervention using both targeted and chemotherapeutics. LBx may additionally identify those patients who have occult secondary tumors as evidenced by persistence of LEVs following primary surgery or predict whether post-operative patients are more or less likely to benefit from adjuvant radiotherapy. For patients at risk of breast cancer, LBx may also have a role as an adjunct to radiologic screening for breast cancer by stratifying the Breast Imaging-Reporting and Data System (BI-RADS) category 3 patients into categories 2 or 4 based on LBx results. Such a combined approach may reduce the patient anxiety associated with indeterminate mammography results and reduce the need for 6 months call-back imaging. Each of these hypotheses require testing in large-scale prospective trials.

## Methods

### Study design

A total of 100 BC patients and 30 normal donors are included in this study. Cancer patients were recruited to the prospective Physical Sciences in Oncology study (PSOC-0068) entitled OPTImization of blood COLLection (OPTICOLL)^68^. Here, we present a subset consisting of 74 patients clinically classified as early-stage and 26 patients clinically classified as late-stage BC at time of enrollment (Supplementary Table 1.). All cancer patients were enrolled between April 2013 and January 17, 2017, at multiple clinical sites in the United States: Billings Clinic (Billings, MT), Duke University Cancer Institute (Durham, NC), City of Hope Comprehensive Cancer Center (Duarte, CA), and University of Southern California Norris Comprehensive Cancer Center (Los Angeles, CA). Patient recruitment took place according to an institutional review board-approved protocol at each site and all study participants provided written informed consent^68,69^. This study was approved by the University of Southern California, University Park Institutional Review Board (FWA 00007099, USC UPIRB #UP-14-00523).

The study schedules were coordinated and unified across the clinical sites. For patients included in this study with non-metastatic treatment naïve disease (early-stage BC), the blood draws were acquired prior to any treatment. Patients with metastatic disease (late-stage BC) had multiple blood specimens collected at the beginning of a new line of therapy, either as a first line of therapy or post-progression while on therapy for the treatment of metastatic malignancy. A total of 10 normal blood donor samples were procured from the Scripps Clinic Normal Blood Donor Service and defined as individuals with no known pathology. Additionally, 20 age and gender-matched normal donor samples were provided from Epic Sciences and defined as women between 45 and 82 yrs (median = 57) with no known pathology. Normal donors will refer to the accumulation of both Scripps Clinic and Epic Sciences samples.

### Blood collection and processing

Approximately 8 mL peripheral blood was collected in 10-mL blood collection tubes (Cell-free DNA BCT, Streck) at the respective clinical site. Blood specimens were shipped to and processed at the Convergent Science Institute in Cancer (CSI-Cancer) at the University of Southern California within 24–48 h of collection, as previously described^20^. Upon receipt, all samples underwent red blood cell lysis and the remaining nucleated cell population was plated in a monolayer on custom-made cell adhesive glass slides (Marienfeld, Lauda, Germany), at approximately 3 million cells per slide. The prepped slides were subsequently incubated in 7% BSA, dried and stored at −80 °C^27,68,69^.

### Immunofluorescence assay

Two slides from each patient, corresponding to approximately 6 million nucleated cells, were thawed and subsequently stained using IntelliPATH FLX™ autostainer (Biocare Medical LLC, Irvine, CA, USA) in batches of 50 slides (46 patient slides [2 slides per patient] and 4 control slides) as previously described^27,44,69^. All steps were performed at room temperature. Cells were fixed with 2% neutral buffered formalin solution (VWR, San Dimas, CA) for 20 min, nonspecific binding sites were blocked with 10% goat serum (Millipore, Billerica, MA) for 20 min. Slides were subsequently incubated with 2.5 ug/mL of mouse antihuman CD31 monoclonal antibody (Ab) (clone: WM59, MCA1738A647, BioRad, Hercules, CA) preincubated with 100ug/mL of goat antimouse IgG monoclonal Fab fragments (115-007-003, Jackson ImmunoResearch, West Grove, PA) for 4 h. After incubation with CD31-Fabs, cells were permeabilized using 100% cold methanol for 5 min. Cells were then incubated with an Ab cocktail consisting of mouse antihuman pan-cytokeratin (PanCK) mAbs (clones: C11, PCK-26, CY-90, KS-1A3, M20, A53-B/A2, C2562, Sigma, St. Louis, MO), mouse antihuman CK19 mAb (clone: RCK108, GA61561-2, Dako, Carpinteria, CA), mouse antihuman CD45 Alexa Fluor® 647 mAb (clone: F10-89-4, MCA87A647, AbD serotec, Raleigh, NC), and rabbit anti-human vimentin (VIM) mAb (clone: D21H3, 9854BC, Cell Signalling, Danvers, MA) for 2 h. Slides were then incubated with Alexa Fluor® 555 goat antimouse IgG1 antibody (A21127, Invitrogen, Carlsbad, CA) and counterstained with 4′,6 diamidino-2-phenylindole (D1306, ThermoFisher, Waltham, MA) for 40 min. Slides were then mounted with an aqueous mounting media to preserve cellular integrity for further downstream analysis.

### Image acquisition and feature extraction

After staining, the slides were imaged using automated high-throughput fluorescence scanning microscopy at 100x magnification, resulting in 2304 image frames per slide, as previously reported^27^. Exposure times and gain for PanCK, VIM, CD45/CD31, and DAPI (DNA) channels were determined computationally by the scanner control software to normalize the background intensity levels across all slides. Using customized EBImage (4.12.2) software and the R scripting language for image analysis, cells were segmented, and their cellular and nuclear descriptors were extracted as previously described^44^.

### Rare event identification, classification, and analysis

**Fig. 6:**
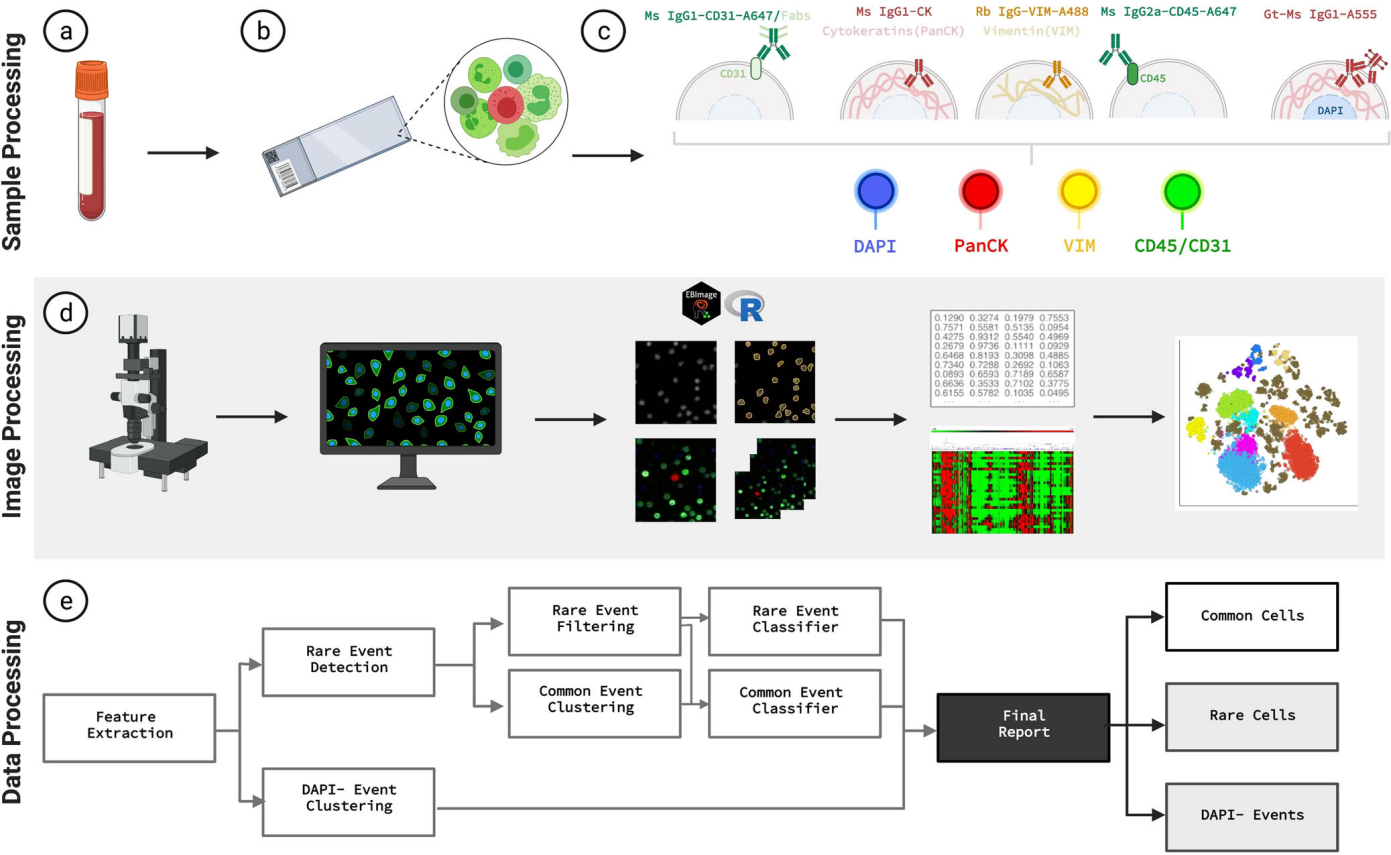
HDSCA3.0 Workflow Overview. **a–c** Blood specimens are collected, processed, and plated onto slides, and undergo immunofluorescent staining. **d** Slides are scanned, acquired images are segmented, cellular features are extracted using R and EBImage software, dimensionality reduction analysis is applied to the cells. **e** Data processing pipeline allows for rare cell detection, filtering, and classification, and DAPI- event separation for curation of final report. Created with BioRender.com.

Rare events were detected by the third-generation of our computational algorithm for unsupervised clustering, as previously described^34^. In brief, this approach allows for the classification of cells into common and rare groups based on principal component analysis of cells‘ morphometric features and subsequent hierarchical clustering (Fig. 6). Additionally, the algorithm identified large DAPI- | PanCK+ events (1–10 µm in diameter) to be classified as LEV candidates, as previously demonstrated^53^.

Rare cells were then further classified into 8 classes based on the combinations of immunofluorescent marker expression in 3 categories: PanCK, VIM, CD45/CD31. Four categories showed no expression of cytokeratins but were determined positive for either VIM or CD45/CD31, or determined positive or negative for both. Enumerations of the cellular categories were done by trained analysts who determined the final enumeration per cell type.

Finally, the frequency of rare events (CTCs and LEVs) for each category was reported as the concentration of rare cells per ml (mean, median, range), calculated by measuring the total number of nucleated cells per two slides, estimated using DAPI-stained nuclei count, against the total complete blood count of the received sample.

### Morphometric comparison

The computational approach uses EBImage to segment cells and extract quantitative cellular and nuclear features^44^. For our morphometric analysis, we utilized the extracted features to further analyze the identified rare cells. Features correspond to cell size and eccentricity, nucleus size and eccentricity, immunofluorescent intensity of the DAPI, PanCK, VIM, CD45/CD31 channels, and the ratios of all combinations of these features to one another. Values for the immunofluorescent channels are reported as the mean signal over cell area, normalized per slide to interval 0-1.

### Statistical analysis

Statistical two-sided analyses were performed using R (Version 4.1.1., Boston, MA). Groups were compared using Kruskal-Wallis (one-way ANOVA on ranks) for non-parametric rank-based dependence between multiple groups to compare whether the distributions have a median shift greater than the null hypothesis, and student’s t-test to determine if there is a significant difference between the means of two groups, for all analyses. *P* values below 0.05 were considered statistically significant. No correction was conducted as the comparisons were *planned comparisons*. Pearson correlation was used to evaluate the relationship between study groups.

### Machine learning model

The primary goal of this study was to determine the ability of HDSCA3.0 rare cell detection to stratify normal donor, early-stage BC, and late-stage BC into distinct groups based on the rare cellular events detected using the LBx approach. While this stratification was initially performed using statistical analysis on the cell counts, we explored the ability of using machine learning models with the target variable of disease state. We used the manual enumeration recorded as event counts per ml per fluorescent channel type. To overcome discrepancies in the sample size, we randomly oversampled the late-stage BC group to match the size of the early-stage BC cohort. Similarly, we oversampled the normal group to match the size of the combined BC groups. To ensure we were not biasing the dataset by oversampling two groups, we also performed combinations of random undersampling of early-stage and oversampling normal, as well as undersampling both early- and late-stage groups.

For the model, we tested random forest, logistic regression, and naïve bayes algorithms using Python 3 (Python Software Foundation, https://www.python.org/) and Orange 3.0 data-mining toolbox in Python^73^. Model comparison was done by measuring the accuracy, sensitivity, specificity, and AUC (area under the ROC curve) to evaluate performance. In all comparisons, the random forest was the top performing algorithm.

To determine the stratification efficiency of the LBx using HDSCA3.0, a random forest algorithm was used to develop models to predict disease state classification. We built a random forest model with 10 trees. Our random forest model was trained, validated, and tested using data from 296 samples (74 early-stage, 74 latestage, and 148 normal donors). Training and validation of the model was performed on ~75% of the dataset through random selection (111 BC and 111 normal donors for cancer vs. normal/56 early-stage BC and 55 latestage BC for early vs. late), using 10-fold cross validation. Testing of the model was performed on the remaining ~25% of the dataset (37 BC and 37 normal donors for cancer vs. normal/18 early-stage BC and 19 late-stage BC for early vs. late), thereby maintaining the class distribution across training/validation/test sets.

## Supplementary information

Supplementary Material

## Data Availability

All data discussed in this manuscript are either included in the main manuscript text. Data files and image repository can be accessed through BloodPAC Accession ID: BPDC000126 and the permalink (URL) https://data.bloodpac.org/discovery/BPDC000126.

## Code availability

The code used to analyze the single data uses standard third-party open-source libraries and packages in R and Python. The code used to identify CTCs in the HDSCA workflow uses custom code and is proprietary and licensed to Epic Sciences for commercial use.
