# Supplementary material for: Multianalyte liquid biopsy to aid the diagnostic workup of breast cancer
Source: NPJ Breast Cancer. 2022 Sep 27;8:112. doi: 10.1038/s41523-022-00480-4 (PMC9515081; doi:10.1038/s41523-022-00480-4)
Supplement: Supplementary file 1 — Supplementary Material [file 41523_2022_480_MOESM1_ESM.pdf]

Supplementary Table 1

	Patients	Early Stage n	Early Stage %	Late Stage n	Late Stage %
Sex	Female	74	100	26	100
	Male	0	0	0	0
	N/A	0	0	0	0
Stage	Early	74	100	0	0
	Late	0	0	26	100
	N/A	0	0	0	0
ER Status	Positive	47	64	9	35
	Negative	6	8	6	23
	N/A	21	28	11	42
PR Status	Positive	43	58	6	23
	Negative	11	15	9	35
	N/A	20	27	11	42
HER2 Status	Positive	7	9.5	4	15
	Negative	40	54	10	39
	N/A	27	36.5	12	46
Histological Subtypes	Ductal	48	58	11	23
	Lobular	5	15	3	35
	Other	1	27	0	42
	N/A	20	27	12	42
Tumor Grade	1	24	32.5	1	4
	2	21	28.5	6	23
	3	9	12	5	19
	N/A	20	27	14	54

N/A: data not provided.

Supplementary Figure 1

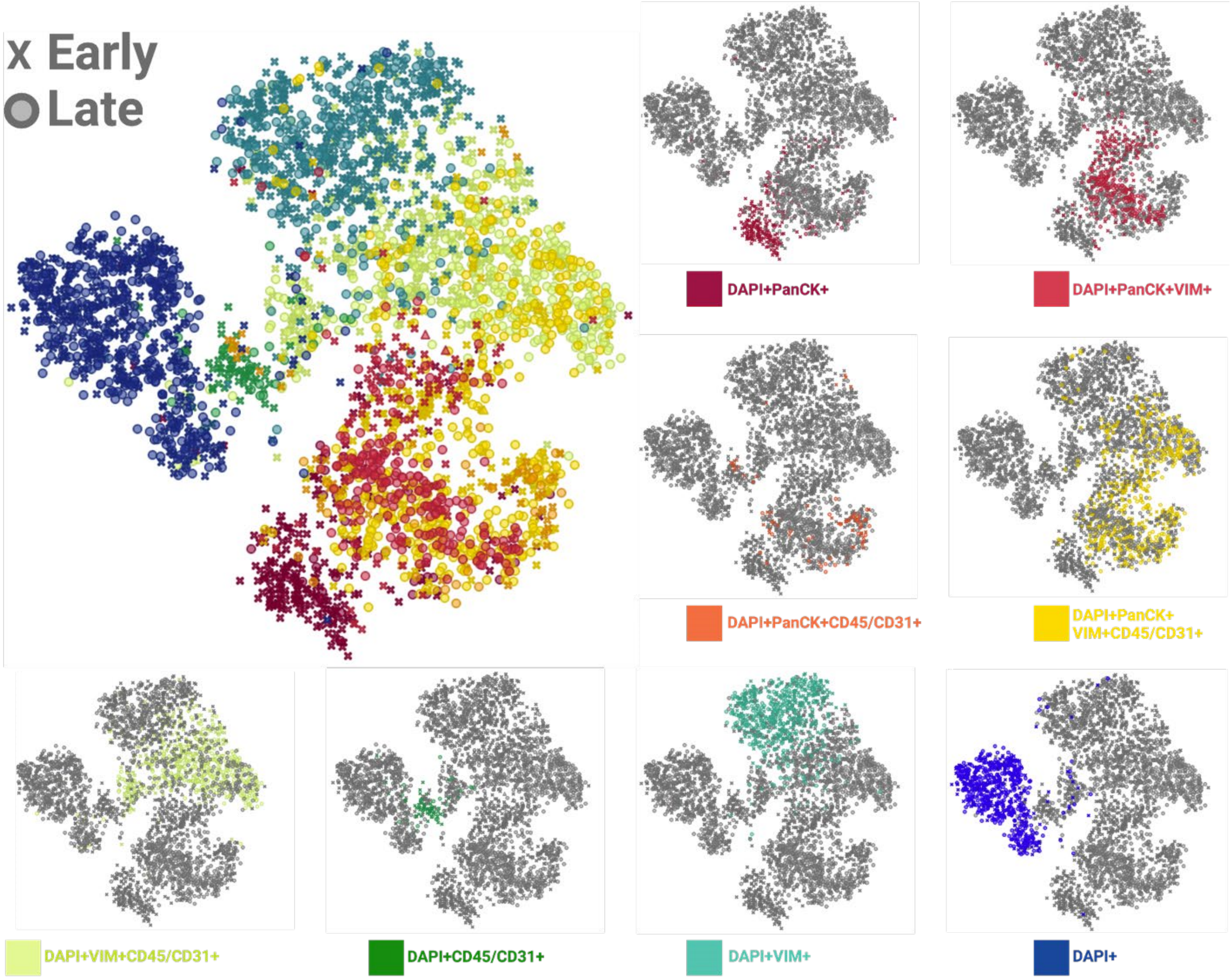

Supplementary Figure 1. Visualization of rare cell features. T-SNE embedding of rare cells based on morphometric features derived from OCULAR. Each designated color represents a classification group marked in the figure.
